# External lysis of *Escherichia coli* by a bacteriophage endolysin modified with hydrophobic amino acids

**DOI:** 10.1186/s13568-019-0838-x

**Published:** 2019-07-15

**Authors:** Guangmou Yan, Rui Yang, Kejia Fan, Hanlin Dong, Chencheng Gao, Shuang Wang, Ling Yu, Zhe Cheng, Liancheng Lei

**Affiliations:** 1 College of Veterinary Medicine, Jilin University, Xi’an Road 5333, Changchun, 130062 Jilin People’s Republic of China; 2 College of Animal Science and Veterinary Medicine, Henan Institute of Science and Technology, Xinxiang, People’s Republic of China

**Keywords:** Antimicrobial substance, Bacteriophage endolysin, *Escherichia coli*, Gram-negative bacteria

## Abstract

Drug-resistant bacteria are a serious threat to global public health. Gram-positive bacterial endolysin preparations have been successfully used to fight Gram-positive bacteria as a novel antimicrobial replacement strategy. However, Gram-negative bacterial phage endolysins cannot be applied directly to destroy Gram-negative strains due to the externally inaccessible peptidoglycan layer of the cell wall; this has seriously hampered the development of endolysin-like antibiotics against Gram-negative bacteria. In this study, 3–12 hydrophobic amino acids were successively added to the C-terminus of *Escherichia coli* phage endolysin Lysep3 to create five different hydrophobic-modified endolysins. Compared with endogenous Lysep3, endolysins modified with hydrophobic amino acids surprisingly could kill *E. coli* from outside of the cell at the appropriate pH and endolysin concentration. The lysis ability of modified endolysins were enhanced with increasing numbers of hydrophobic amino acids at the C-terminus of endolysin. Thus, these findings demonstrate that the enhancement of hydrophobicity at the C-terminus enables the endolysin to act upon *E. coli* from the outside, representing a novel method of lysing Gram-negative antibiotic-resistant bacteria.

## Introduction

The excessive use of antibiotics in clinical medicine and raising livestock have resulted in an increased prevalence of antibiotic-resistant strains (Morrill et al. 2016). The ability of bacteria to resist antibiotics has been spread and strengthened through naturally occurring plasmids and bacteriophages. The problem of antibiotic resistance is worsening and represents a grave threat to human health (Tacconelli et al. 2018). Phage endolysins can efficiently, rapidly, and specifically lyse drug-resistant pathogens without affecting the commensal flora (Yang et al. 2014). Moreover, bacteria are less likely to develop resistance to endolysins. Therefore, increased attention has recently been paid to studies of phage endolysins as potential therapies (Gerstmans et al. 2016; Rodríguez-Rubio et al. 2017).

There is now evidence that lysin preparations can kill Gram-positive bacteria, such as *Streptococcus pneumoniae* and *Staphylococcus aureus*, both in vivo and in vitro (Loeffler et al. 2001; Nelson et al. 2001; Schuch et al. 2002; Schmelcher et al. 2012). Although lysin preparations have entered phase II clinical trials in Gram-positive bacterial infection, the outer membrane protein pore size of Gram-negative bacteria only allows small molecules to pass through. Endolysins cannot pass through and reach the inner peptidoglycans layer in the periplasmic space of Gram-negative bacteria (Nikaido 2003). Thus, current endolysin formulation are unsuitable for clinical use.

Recently, several natural or genetically engineered endolysins have been reported to cleave Gram-negative bacteria (Lai et al. 2011; Walmagh et al. 2012; Briers et al. 2014; Lood et al. 2015; Guo et al. 2017). We compared and analyzed the molecular structures of these endolysins, and found abundant charges and hydrophobic amino acids at their ends. We hypothesized that the ability of these proteins to pass through the outer membrane of bacterium was associated with the number of charges and hydrophobic amino acids. A previous study found that the addition of the positive charge at the C-terminal of endolysins could make the endolysins effectively lyse the *E. coli* from the outside of the cell (Ma et al. 2017). However, whether the hydrophobic amino acids could affect the lytic effect from the outside of the cell was still unknown.

Therefore, the purpose of this study was to explore the effect of hydrophobicity at the C-terminus of endolysins on the cleavage of Gram-negative bacteria and provide a novel method of modifying endolysins for use against antibiotic-resistant bacteria.

## Materials and methods

### Strains and materials

In this study, the *E. coli* strains DH5α and BL21 (DE3) (Takara, Dalian, China) were used for cloning and expressing the fusion endolysins, respectively. *E. coli* strains (ATCC25922, CVCC1418 and O78), *Acinetobacter baumannii* (*A. baumannii*) strain, and *Streptococcus suis* (*S. suis*) strain were stored in the lab. The restriction endonucleases *Nde*I and *Sal*I, T4 DNA ligase, and pET-28a vector (Takara, Dalian, China) were used to construct the recombinant vectors. Genscript Ni–NTA affinity chromatography medium (GE Healthcare, Beijing, China) was used for purifying endolysins, and primers were synthesized by Jilin Ku Mei Biotechnology Co., Ltd.

### Design of fusion endolysins

Lysep3 (Gene bank: 22112958), a coliphage endolysin that was previously isolated by our lab (Lv et al. 2015), is unable to cleave *E. coli* from outside the cell (Yan et al. 2017). The hydrophobic amino acids Ile and Leu were selected among Ile, Val, Leu, Phe, and Ala (hydrophobic parameters: 4.5, 4.2, 3.8, 2.8, and 1.8, respectively) to be added to the C-terminal of Lysep3. Additionally, Pro was added to facilitate expression in prokaryotes, and the product was named Lysep3-3. The hydrophobicity index of Lysep3-3 was predicted by ExPASy web server to be 1.711. Five hydrophobic amino acids (Phe, Phe, Val, Ala, Pro) were added to the C-terminal of Lysep3 to construct the fusion endolysin Lysep3-5, which had a hydrophobicity index of 2.311. Additionally, we added 7 hydrophobic amino acids (Phe, Val, Phe, Ile, Phe, Ala, and Pro), 12 hydrophobic amino acids (Phe, Ile, Leu, Ile, Val, Phe, Val, Leu, Ile, Ile, Ala and, Pro), and 12 hydrophobic amino acids (Phe, Ile, Val, Ile, Leu, Ile, Val, Phe, Leu, Ile, Ala, and Pro) to the C-terminal of Lysep3; these fusion endolysins were designated Lysep3-7, Lysep3-12a, and Lysep3-12b with the hydrophobicity index of 2.889, 4.089, and 4.089, respectively. While the hydrophobicity index of Lysep3-12a and Lysep3-12b were the same, their amino acid sequences were different.

### Cloning, expression, and purification of fusion endolysins

The expression and purification of Lysep3 were performed as previously described (Yan et al. 2017). *Lysep3* gene as a template was used for amplifying other fusion endolysins. The common upstream primer (5′-GGAATTCCATATGAAAATTTCATCCAATGGCCT-3′) contained a *Nde*I site and the protection base; downstream primers contain a *Sal*I site. Hydrophobic amino acids were added to the 3′ end of *Lysep3*, as shown in Table 1. Target fragments were amplified with the following reaction conditions: denaturation at 98 °C for 1 min; denaturation at 95 °C for 10 s, annealing at 63 °C for 15 s, and extension at 72 °C for 15 s, for a total of 30 cycles; there was also a final extension at 72 °C for 10 min. Target genes were recovered and digested with *Nde*I and *Sal*I at 37 °C for 30 min. DNA products were re-purified and cloned into plasmid pET-28a to generate the recombinant constructs pET-3, pET-5, pET-7, pET-12a, and pET-12b.

**Table 1:** Sequences of hydrophobic peptide fusion lyase primers

Peptide	Sequence	Notations
3-3	GGAATTCCATATGAAAATTTCATCCAATGGCCT	Sharing upstream primers
	ACGCGTCGACTCACGGCAGAATTGCTGCCGCCACACCGCG	3 hydrophobic peptides added
3-5	ACGCGTCGACTCAGGGAGCAGCGACGAATGCTGCCGCCACACCGCGTT	5 hydrophobic peptides added
3-7	ACGCGTCGACTCATATTATGGGAGCAGCGACCCAGAATGCTGCCGCCACACCGCGTT	7 hydrophobic peptides added
3-12a	ACGCGTCGACTCACGGCGCAATAATCAGCACGAACACAATCAGAATGAATGCTGCCGCCACACCGCGTTCAATAGC	12 hydrophobic peptides added
3-12b	ACGCGTCGACTCACGGCGCAATCAGGTTCACAATCAGAATCACAATGAATGCTGCCGCCACACCGCGTTCAATAGC	12 hydrophobic peptides added

These plasmids were then transformed into BL21 (DE3) to express recombinant proteins. The expression and purification of Lysep3, Lysep3-3, and Lysep3-5 were performed as previously described (Yan et al. 2017).

### Effects of pH on the activity of recombinant endolysins

These experiments were performed as previously described (Yan et al. 2017) with small modifications. A single *E. coli* BL21 (DE3) colony was cultured in 5 mL of LB medium at 37 °C with shaking at 180 rpm until the OD_600 nm_ reached 0.45. We then removed 1 mL of bacterial suspension and diluted it 10^6^-fold. Then, 200 μL of bacterial suspensions were added to 21 sterile 1.5 mL tubes and centrifuged at 12,000 rpm. After discarding the supernatants, 300 μL of phosphate-buffered saline (PBS) at pH 4.5, 5.0, 5.5, 6.0, 6.5, 7.0, and 7.5 were added to seven sterile tubes as control treatments. For another seven tubes, 206 μL of PBS at pH 4.5, 5.0, 5.5, 6.0, 6.5, 7.0, and 7.5 was individually added into sterile tubes, and then 94 μL of Lysep3-5 (original concentration: 5.59 μg μL^−1^, final concentration: 1.75 μg μL^−1^) was added to each tube and mixed well. Lysep3 was added in the same manner to the final seven tubes (original concentration: 5.59 μg μL^−1^, final concentration: 1.75 μg μL^−1^). All tubes were then incubated at room temperature for 30 min. Finally, 200 μL of bacterial suspension was cultured on solid LB medium at 37 °C for 16 h, and bacterial colony numbers were determined. Each group was tested three times.

### Effects of concentration on the lytic ability of fusion endolysins

The cultivated BL21 (DE3) were diluted 10^6^-fold using the methods described above. We added 150 μL of bacterial suspension to 11 tubes, then centrifuged the tubes at 12,000 rpm and discarded the supernatants. Next, 246, 233, 220, 206, or 193 μL of PBS at pH 5.0 were added to resuspend the bacterial pellets, to which was added 54, 67, 80, 94, or 108 μL of Lysep3-5 (5.59 μg μL^−1^), respectively, so that the concentration of Lysep3-5 was 1.0, 1.25, 1.5, 1.75, or 2.00 μg μL^−1^, respectively, before incubation at room temperature for 30 min. The 100 μL of bacterial suspension were then cultured on solid LB medium, and bacterial colony numbers were determined. Lysep3 was added to the other five tubes for comparison, with concentrations of 1, 1.25, 1.5, 1.75, and 2 μg μL^−1^. Each group was tested three times, and 300 μL of PBS at pH 5.0 was used as the control treatment.

### Effects of different numbers of hydrophobic amino acids on the lytic ability of fusion endolysins

A single *E. coli* BL21 (DE3) colony was cultured in 5 mL of LB medium at 37 °C with shaking at 180 rpm until the OD_600 nm_ reached 0.45. Then, 1 mL of bacterial suspension was diluted 10^6^-fold. Subsequently, 150 μL of diluted bacterial suspension was transferred into 1.5 mL tubes, centrifuged at 12,000 rpm, and then the supernatants were discarded. A total of 206, 199, 206, 24, 95, and 120 μL of PBS at pH 5.0 were added to the tubes, as well as 94, 101, 94, 276, 205, and 180 μL of Lysep3, Lysep3-3, Lysep3-5, Lysep3-7, Lysep3-12a, and Lysep3-12b, respectively, to a final concentration of 1.75 μg μL^−1^. Tubes were then incubated at room temperature for 30 min. Finally, 100 μL of bacterial suspension was cultured on solid LB medium at 37 °C for 14 h, and bacterial colony numbers were determined. Each group was tested three times.

### Effects of EDTA on the lytic ability of fusion endolysins

Equal amounts of bacteria, final concentration 1.75 μg μL^−1^ of Lysep3, and fusion endolysins were added to 1.5-mL centrifuge tubes, and then 25 μL of ethylenediaminetetraacetic acid disodium salt (EDTA) at 25 mM (final concentration: 0.5 mM) was added to each tube. All tubes were incubated at room temperature for 30 min. Finally, 100 μL of bacterial suspension was cultured on solid LB medium at 37 °C for 14 h, and bacterial colony numbers were determined. Each treatment was tested three times.

### Detection of the bacteriostatic spectrum

The cultivated 100 μL of *E. coli* strains (ATCC25922, CVCC1418, DH5α, O78), *A. baumannii* strain, and *S. suis* strain each was diluted 10^6^-fold. Then, 150 μL of each strain was picked out and added into a tube, total three tubes. The bacteria suspensions were centrifuged at 12,000 rpm for 1 min, and the supernatants were discarded. Each strain was treated with PBS, Lysep3, and Lysep3-5 (final concentration 1.75 μg μL^−1^), and a final volume of 300 μL was reached with PBS (pH 5.0). After the treatment, the bacterial colonies were counted using the methods described earlier.

### Statistical analysis

For statistical analyses, one-way ANOVA was performed using SPSS software (v12.0; SPSS Inc., Chicago, IL, USA). P-values < 0.05 were considered statistically significant.

## Results

### Design, expression, and purification of fusion endolysins

**Fig. 1:**
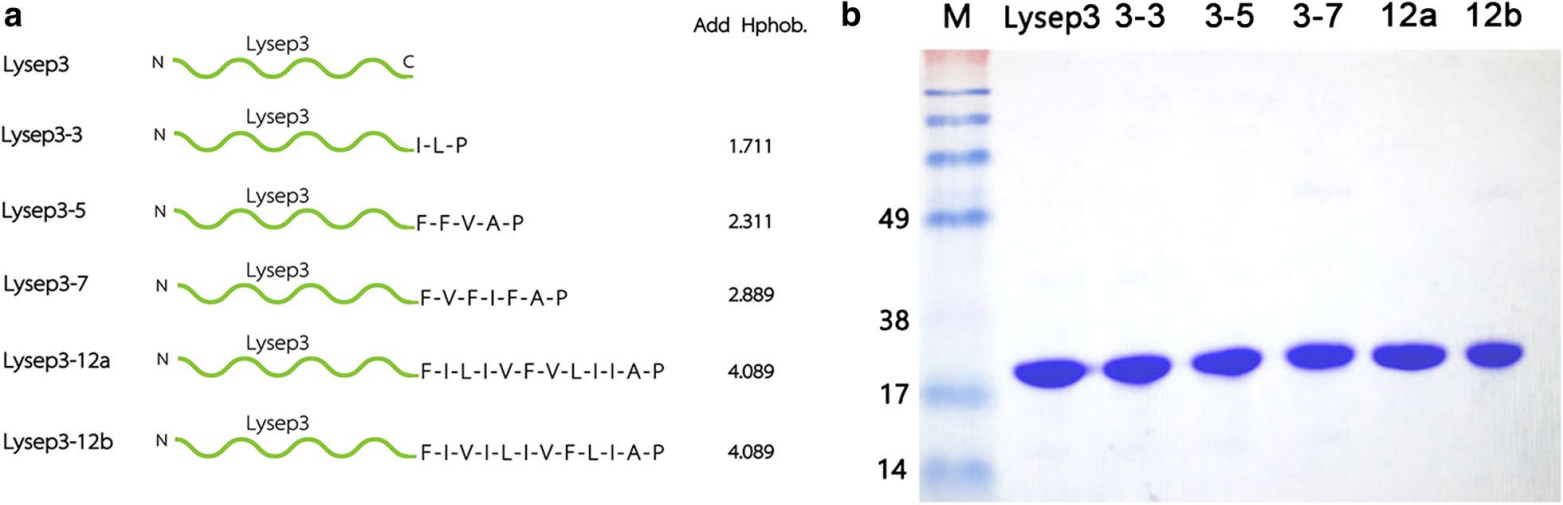
Design and expression of fusion endolysins. **a** The schematic diagram of the structures of fusion endolysins. N and C represent the N-terminal and C-terminal of Lysep3, respectively. I (Ile), L (Leu), P (Pro), F (Phe), V (Val), and A (Ala). **b** The SDS-PAGE results of purified fusion endolysins. The first lane is a 180-kDa protein marker, and the latter lanes are Lysep3 (17.7 kDa), Lysep3-3 (18.3 kDa), Lysep3-5 (18.9 kDa), Lysep3-7 (19.5 kDa), Lysep3-12a (18.9 kDa), and Lysep3-12b (18.9 kDa)

To explore the effect of hydrophobic amino acids on the ability of endolysins to get into the outer membrane of Gram-negative bacteria, different hydrophobic amino acids were added to the C-terminus of Lysep3, generating the constructs: Lysep3, Lysep3-3, Lysep3-5, Lysep3-7, Lysep3-12a, and Lysep3-12b (Fig. 1a). These endolysins were then expressed and purified, and SDS-PAGE results showed that their purity reached up to 98.7–99.3% (Fig. 1b).

### The effect of pH and concentration on lysis activity

A previous study found that adding three hydrophobic amino acids to the C-terminus of Lysep3 endowed the enzyme with the ability to lyse *E. coli* from outside, but bactericidal effects were not obvious. However, when five hydrophobic amino acids were added, a significant bactericidal activity was observed. Therefore, Lysep3-5 was used as a representative endolysin and Lysep3 as the control to determine the most effective conditions for endolysin-mediated bacterial cleavage.

**Fig. 2:**
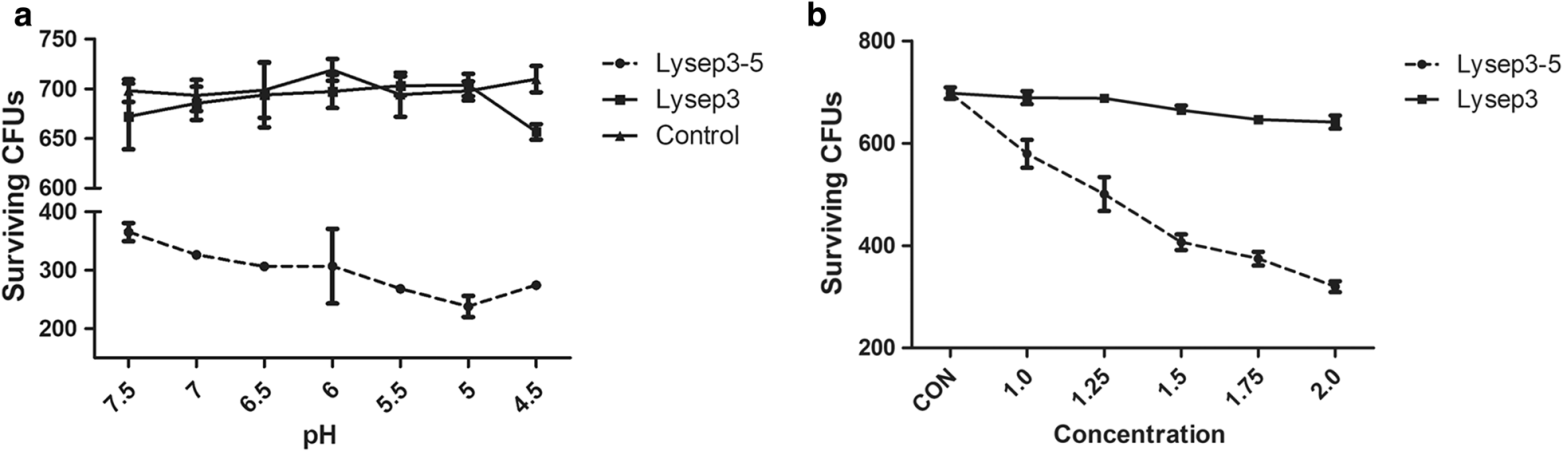
Lysis activity of Lysep3 and Lysep3-5 at different pH values and concentrations. **a** The effect at different pH values on bacteria survival. **b** The bactericidal effect at different concentrations (μg μL^−1^) of Lysep3 and Lysep3-5. The Lysep3 and Lysep3-5 groups originally had the same bacteria concentrations

First, the optimal pH was determined, at which the fusion endolysins most effectively lysed bacteria. The results showed that the lysis activity of Lysep3 was no different with the PBS control group (P > 0.05). However, compared with the PBS and Lysep3 groups, Lysep3-5 significantly lysed bacteria (P < 0.05). Moreover, Lysep3-5 had the strongest lysis activity at pH 5.0 (Fig. 2a), which was similar to a previously engineered fusion endolysin (Yan et al. 2017). Thus, this modified endolysin could effectively lyse host bacteria at pH 5.0.

Next, we examined lysis activity at different endolysin concentrations at pH 5.0. These results showed that different Lysep3 concentrations did not lead to bacterial lysis; however, Lysep3-5 significantly decreased bacterial colony numbers. Additionally, higher Lysep3-5 concentrations were associated with improved bactericidal effects. The bactericidal capacity of the modified endolysin showed maximal activity at 1.75 μg μL^−1^, and there was no significant difference in bacterial lysis activity at concentrations > 1.75 μg μL^−1^ (Fig. 2b). These results indicated that the modified endolysin had the most effectively bactericidal effect at 1.75 µg μL^−1^ and at pH 5.0.

### Increased numbers of hydrophobic amino acids improved bactericidal ability

**Fig. 3:**
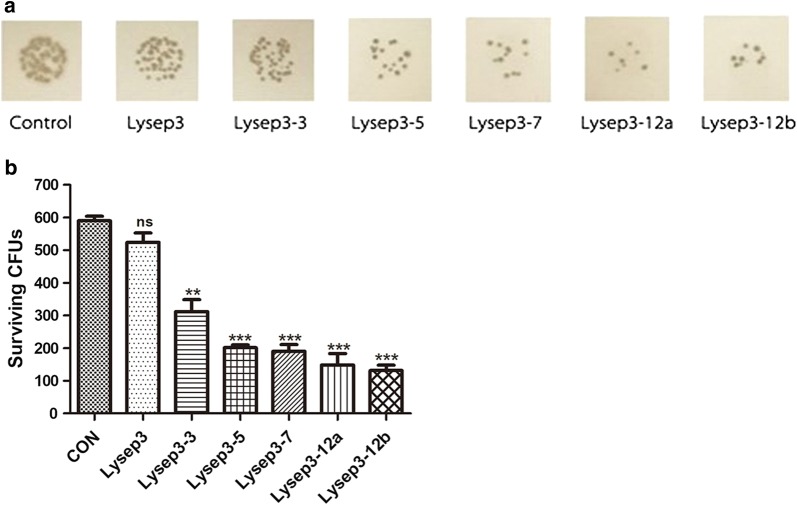
Bacterial growth on a solid LB medium. **a** After incubation with different fusion endolysins, bacteria mixtures were dropped onto a solid LB medium and cultured. **b** Bacteria were diluted, incubated with endolysins, and plated on a solid LB medium. Then, colonies were counted after culture. ns, no significance; *P < 0.05; **/***P < 0.01

Next, we investigated the effect of different numbers of C-terminal hydrophobic amino acids on bactericidal ability. Lysep3, Lysep3-3, Lysep3-5, Lysep3-7, Lysep3-12a, and Lysep3-12b were diluted to 1.75 µg μL^−1^, and their bactericidal ability was compared at pH 5.0. After the bacteria were treated, the mixture was dripped onto solid LB medium. The results showed that Lysep3-3, Lysep3-5, Lysep3-7, and Lysep12 had smaller and fewer bacterial colonies. Moreover, the more hydrophobic amino acids that were added, the stronger the bactericidal effect. Additionally, there was no difference between Lysep12a and Lysep12b, which have the same hydrophobic index but different C-terminal amino acid sequences (Fig. 3a). Furthermore, colony counts explain this result in more detail. Briefly, bacteria were diluted, incubated with endolysins, and counted. The results showed that the Lysep3 group had the same number of bacteria as the PBS control group (P > 0.05). Compared with the PBS control and Lysep3 groups, the other groups had fewer bacterial colonies (P < 0.05) (Fig. 3b). These results indicated that increased numbers of hydrophobic amino acids could strengthen the bactericidal ability of endolysins.

### EDTA strengthened the bactericidal ability of endolysin

**Fig. 4:**
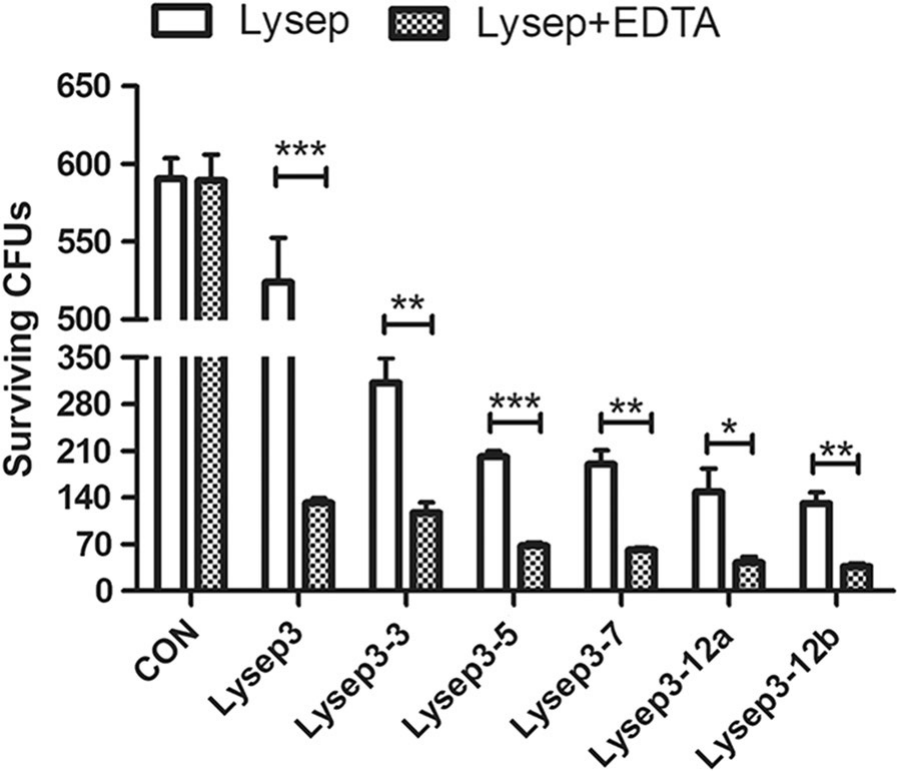
Effects of EDTA on the bactericidal performance of fusion endolysins. Comparison of the bactericidal properties of fusion endolysins (final concentration 1.75 μg μL^−1^) in the presence of EDTA. ns, no significance; *P < 0.05; **/***P < 0.01

EDTA has been shown to improve the ability of bacteriophage endolysins to cleave Gram-negative bacteria in vitro (Wang et al. 2017). Therefore, we investigated the effect of EDTA on the lysis activity of modified endolysin. The results showed that there was no difference between the two PBS control groups, which indicated that EDTA itself had no bactericidal effect. However, the bactericidal effect of Lysep3 was increased after EDTA treatment, which allowed Lysep3 to lyse bacteria from the outside; this was also observed in the other groups (Fig. 4). Again, there was no difference between the Lysep12a and Lysep12b groups (Fig. 4).

### Bacteriostatic spectrum of fusion endolysin

**Fig. 5:**
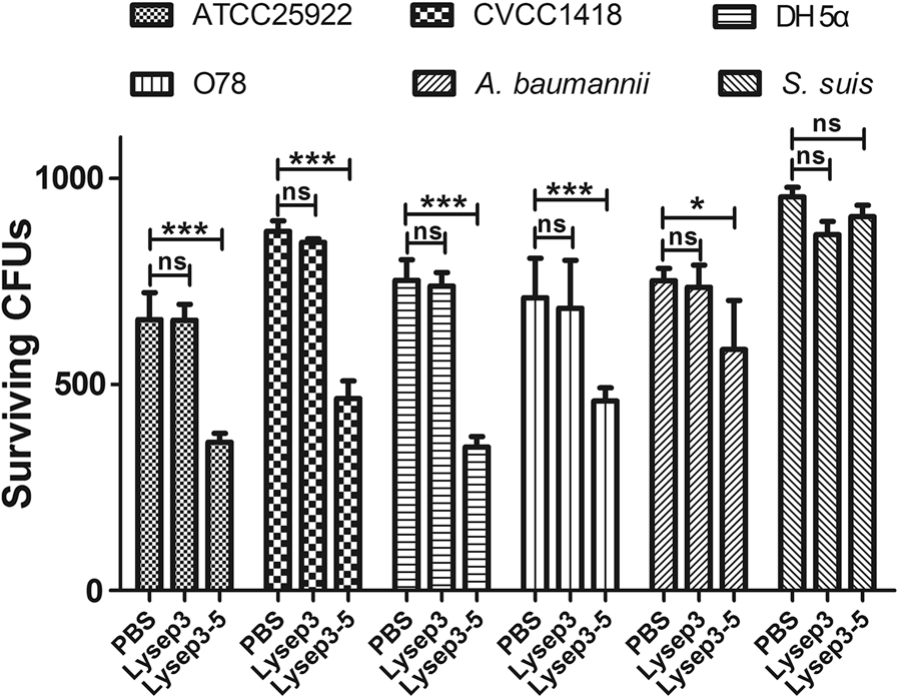
Detection of the bacteriostatic spectrum. *E. coli* strains (ATCC25922, CVCC1418, DH5α, and O78), *Acinetobacter baumannii* strain, and *Streptococcus suis* strain were treated with PBS, Lysep3, and Lysep3-5, and the colonies were counted. ns, no significance; *P < 0.05; **/***P < 0.01

Next, the lytic effects of lysin on other Gram-positive and Gram-negative bacteria were examined. The result showed no difference between the PBS and Lysep3 groups (P > 0.05). Compared with PBS and Lysep3 groups, Lysep3-5 had a prominent bactericidal effect on *E. coli* strains ATCC25922, CVCC1418, DH5α, and O78 (P < 0.01). In addition, it had a certain bactericidal effect on *A. baumannii* (P < 0.05). However, it had no bactericidal effect on Gram-positive *S. suis* (P > 0.05) (Fig. 5). Therefore, the modification of hydrophobic amino acids could make endolysins effectively lyse *E. coli* from the outside of the cell.

## Discussion

Phage endolysins have been used to treat diseases caused by Gram-positive bacteria. However, due to the special structure of the cell wall in Gram-negative bacteria, phage endolysins cannot lyse the bacteria from the outside of the cell, which is not the same with the Gram-positive bacteria. This problem limits the application of endolysins. In a previous study, the endolysin could kill the Gram-negative bacteria from the outside by altering the charge number at the C-terminal of the endolysin (Ma et al. 2017). However, whether hydrophobicity has the same effect needs to be further studied. Therefore, the endolysins were modified with hydrophobic amino acids at the C-terminal, and different endolysins were expressed in the prokaryotic expression system. The lysis ability was detected at different pH values and endolysin concentrations. The results showed that the enhancement of hydrophobicity at the C-terminal enabled the endolysin to damage *E. coli* from the outside of the cell wall at pH 5.0 and concentration of 1.75 µg μL^−1^. Moreover, the lysis activity could be strengthened by EDTA. The fusion endolysins could effectively lyse different *E. coli* strains from the outside of the cell. It might bring a novel method to fight with Gram-negative antibiotic-resistant bacterial infection.

The modification of phage endolysin by gene engineering to lyse Gram-negative bacteria is a novel and effective strategy. Wang et al. fused the Lysep3 with the cell wall binding domain D8, and the fusion endolysin Lysep3-D8 could lyse the host *E. coli* from the outside of the cell (Wang et al. 2017). The Lysep3 was also fused with the Colicin A, which could transport the endolysin into the periplasmic space of the cell wall and make the endolysin effectively lyse the bacteria (Yan et al. 2017). However, nothing is perfect. The molecular mechanism of lysing host bacteria is still unknown. Moreover, the D8 and Colicin A have large molecular weights, resulting in inconvenience in their expression and production and hence making them less cost-effective. Ma et al. modified the Lysep3 by altering the number of charges at the C-terminal to make the fusion endolysin effectively lyse the host bacteria (Ma et al. 2017). However, its bactericidal ability may be affected by the pH value in the environment. Compared with that, the restricted conditions of hydrophobic amino acids may be less, making them more cost-effective and easier to produce and apply in practice.

This study found that increased numbers of hydrophobic amino acids could strengthen the bactericidal ability of endolysins. However, no differences were found between the Lysep12a and Lysep12b groups, although they had different amino acid sequences (Fig. 3). It might be because they had the same hydrophobic index, indicating that hydrophobicity could play a main role in the lytic effect and might have nothing to do with the amino acid sequence. This needs further investigation.

*Escherichia coli* is a representative Gram-negative bacterium with a similar cell wall structure as other Gram-negative bacteria. In a way, it is the optimal model for studying phage endolysins in the laboratory. Therefore, endolysins were modified with hydrophobic amino acids and found to effectively kill *E. coli* from the outside of the cell. This indicated that modified endolysins could also lyse other Gram-negative bacteria, which would require further study, but could be a meaningful discovery. This study has important significance for the modification of endolysins and their application in clinical practice.

## Data Availability

All data generated or analyzed during this study are authentic and believable.
